# Moving beyond dashboards to generate data for public good

**DOI:** 10.1016/j.lansea.2022.100047

**Published:** 2022-07-27

**Authors:** Tushar Garg, Pranab Chatterjee

**Affiliations:** a Department of Epidemiology, Johns Hopkins Bloomberg School of Public Health, Baltimore, USA; b Department of International Health, Johns Hopkins Bloomberg School of Public Health, Baltimore, USA

We commend Yadav et al for integrating multiple data sources into a dashboard using open-source tools for illustrating malaria epidemiology in India.^1^ Their vision is to catalyze malaria elimination efforts by creating a comprehensive and spatially granular database and dashboard that can be used by all stakeholders, including researchers and citizens.^2^ However, they only provide a scaffolding for the dashboard without any underlying data citing “data protection issues.” This limits the potential promise of the dataset.

We offer three suggestions to augment their tool. First, data munging, which involves dataset identification, cleaning, transformation, and compiling, is the primary component in building this dashboard. This should be augmented to a data pipeline that outputs compiled data following the FAIR (Findability, Accessibility, Interoperability, and Reuse of digital assets) principles.^3^ Second, the compiled data should be made publicly available through the Government Open Data License under the government's National Data Sharing and Accessibility Policy.^4^ Third, the authors plan to integrate datasets like the Census and National Family and Health Survey to make it more resourceful. By aligning their efforts with the National Data and Analytics Platform of India, they can leverage diverse public datasets by combining the integrated malaria data and putting the data into the hands of the stakeholders.^5^

In summary, dashboards without public access to the underlying data restrict their utility. Moving beyond dashboards to create a truly open and integrated dataset ecosystem of malaria epidemiology can be a true public good, informing the processes of moving towards the vision of a malaria-free India.

## Contributors

TG wrote the original draft, and both authors contributed to the review and editing.

## Declaration of interests

We have no conflicts of interests to declare.
